# A Critical Narrative Review and Expert Consensus on the Clinical Positioning of Non‐Ablative Laser‐Based Therapy in Acne Treatment

**DOI:** 10.1111/jocd.71218

**Published:** 2026-09-28

**Authors:** Fabienne Ballanger, Marie‐Thérèse Leccia, Nicole Auffret, Jean‐Paul Claudel, Brigitte Dréno

**Affiliations:** ^1^ Private Practice Talence France; ^2^ Department of Dermatology, Allergology and Photobiology, CHU Grenoble Alpes University Grenoble Alpes, Inserm U1209, CNRS UMR5309, Institute for Advanced Biosciences France; ^3^ Private Practice Paris France; ^4^ Private Practice Tours France; ^5^ Immunology and New Concepts in ImmunoTherapy, INCIT, UMR 1302/EMR6001 Nantes Université, INSERM, CNRS Nantes France

**Keywords:** acne, laser, light therapy, treatment

## Abstract

**Introduction:**

Acne is characterized by inflammatory and non‐inflammatory lesions. Traditional therapies include topical and systemic agents, which often face limitations such as resistance and side effects. Non‐ablative laser‐based therapy (LBT) has emerged as a promising modality, offering targeted effects on sebaceous glands and *Cutibacterium acnes*.

**Objectives:**

This work discusses the mechanisms, clinical applications, efficacy, limitations, and future directions of laser therapy in active acne lesion management, excluding scars and post‐inflammatory hyperpigmentation.

**Material and Methods:**

Five experts in acne analyzed the content of 15 published sources between 2004 and 2025 and propose an acne treatment approach that includes the use of non‐ablative LBT.

**Results:**

Limited clinical evidence suggests that non‐ablative LBT provides safe and effective reduction of lesions. However, variability in protocols, limited clinical evidence, and accessibility do not allow confirmation of non‐ablative LBT as a valuable treatment alternative to conventional treatment. Non‐ablative LBT may be safe and effective as an alternative to first‐line intention medication in mild acne and as an adjuvant second‐line treatment to conventional treatment or if conventional second‐line acne treatment fails. Efficacy remains to be confirmed through well‐designed and sufficiently powered clinical studies.

**Conclusion:**

Non‐ablative LBT may be a useful and effective future option for acne management, offering both lesion clearance and cosmetic improvement. Proper protocol selection and validated clinical investigations in large cohorts that allow for a critical assessment of the different available non‐ablative LBT approaches remain mandatory to objectively confirm its clinical benefit in the treatment of active acne lesions.

## 1 Introduction

The pathogenesis of acne is multifactorial and involves hyperkeratinisation, excessive sebum production, and immune‐inflammatory responses [1]. Dysbiosis of the phylotypes of the skin commensal *Cutibacterium acnes* (*C. acnes*) plays an important role in acne with a reduced diversity of its 6 phylotypes and with phylotype I (*C. acnes* IA1) being increased in fraction [2, 3, 4].

Current conventional therapies include topical retinoids, benzoyl peroxide, systemic antibiotics, or zinc salt, or hormonal treatments, and isotretinoin, which remain first‐line treatments according to the severity of acne [5, 6, 7]. However, treatment success is frequently limited by variable responses, treatment‐related side effects, frequent relapses, and poor adherence, as the great majority of acne treatments have suppressive rather than curative effects, as well as concerns about antibiotic resistance [8, 9]. Thus, despite current guideline‐based therapies, a substantial proportion of patients show suboptimal responses or intolerance, highlighting an unmet clinical need for alternative or adjunctive non‐systemic therapies.

In this context, laser‐based therapies (LBT) have emerged as promising adjuncts or alternatives in the treatment of active acne lesions. These modalities offer targeted intervention against pathogenic factors such as sebaceous gland activity, commensal bacteria, especially *C. acnes*, and microvascular inflammation with generally favorable safety profiles [10]. A recently published paper by Ishii et al. confirmed the potential role of LBT, especially non‐ablative, LBT in the treatment of active acne lesions, post‐acne marks, and skin quality, without proposing clear and practical recommendations of their use, describing the mechanism of action, and without describing potential limitations of LBT [11].

The purpose of the present article is to provide a critical analysis of non‐ablative LBT in the treatment of active acne lesions, its mechanisms of action, clinical efficacy, safety concerns, and a proposal for optimizing acne care incorporating non‐ablative LBT.

## 2 Material and Methods

Five experts in acne performed a PubMed and Google Scholar literature analysis. Based on these literature sources, the experts performed a critical narrative review of the use and benefits of non‐ablative LBT for the treatment of active acne lesions.

During the first step, the experts searched and analyzed articles published between January 2004 and March 2026 using PubMed and Google Scholar with search terms including “active acne,” “acne,” “laser‐based therapy,” “fractional” and “non‐fractional laser,” and “laser surgery” alone and combined with “active acne,” “active acne lesions”. The overall research and analysis included clinical studies and reviews but excluded case reports leading to the identification of a total of 73 studies. Of these, 46 articles investigating the efficacy of non‐ablative LBT in active acne lesions, published between 2004 and 2026, were identified and analyzed. A study‐centered literature research approach identified 15 clinical studies using various non‐ablative LBT modalities for the treatment of active acne lesions, which served as a basis to assess the clinical evidence for existing data about non‐ablative LBT for the treatment of these lesions.

During the second step, each expert randomly analyzed three to four articles according to population, acne severity, location, previous and concomitant treatment, type and parameters of non‐ablative LBT, and outcome.

During the third step, the authors assessed the level and relevance of clinical evidence for each article in agreement with the evidence grading of the Oxford center for Evidence‐Based Medicine [12]. According to the outcome, studies were rated and are listed in Table 2.

Moreover, the experts discussed the role and clinical benefit of the different non‐ablative LBT options and amended a currently proposed treatment algorithm by incorporating non‐ablative LBT.

## 3 Results

To date, different types of non‐ablative LBT are available for the treatment of active acne lesions, including non‐fractional and fractional lasers, each with specific mechanisms and clinical targets.

Non‐ablative laser produces dermal thermal injury while preserving the epidermis. Fractional lasers produce microscopic columns of thermal injury in the epidermal and dermal tissue, arranged in regularly spaced arrays and affecting only a fraction of the skin surface, thereby avoiding damage to large areas of the skin [27].

Non‐fractional LBT used to treat active acne lesions includes 1450, and 1726‐nm diode lasers, 1540‐nm Erbium:Glass Laser, pulsed dye laser (PDL), and potassium titanyl phosphate (KTP) laser. Fractional lasers used in acne include 1064‐nm Nd:YAG laser.

They act on the pilosebaceous unit, *C. acnes* and *Staphylococcus epidermidis* ( *S. epidermidis* ) [2, 28]. As sebaceous glands are primarily located in both the mid and deep dermis, the penetration depth of the laser is key. Therefore, mid‐infrared lasers may be considered well suited, as they reach the dermal sebaceous glands positioned roughly 1000 μm beneath the stratum corneum. The mechanisms of non‐ablative LBT differ depending on the laser type and wavelength, allowing tailored treatment approaches.

Table 1 provides a summary of the different non‐fractional and fractional laser types used to treat active acne lesions.

**TABLE 1:** Selection of the currently reported non‐ablative LBT used to treat active acne lesions based on published clinical data.

	Mechanism of action	Efficacy	Local tolerance	Clinical evidence	References
Non‐fractional					
1540‐nm diode laser	disrupts sebaceous gland function	Significant (*p* < 0.05) reduction in lesion count (up to 83%); high long‐term remission rate > 70%	Mild local pain	++	[13, 14]
1726‐nm diode laser	disrupts sebaceous gland function	Significant (*p* < 0.03) reduction in inflammatory lesion count reduction after 12 weeks	Mild discomfort; no serious device‐related adverse effects	++	[15, 16]
595‐nm PDL	disrupts sebaceous gland function; produces phototoxic effects to reduce *C. acnes* load	Significant (*p* < 0.01) reduction of acne lesions and sebum secretion; effective in pigmented lesions	Mild erythema, transient pigmentation changes	++	[17, 18, 20]
589/1319‐nm solid‐state dual‐wavelength laser	disrupts sebaceous gland function; produces phototoxic effects to reduce *C. acnes* load	Substantial reduction in inflammatory lesion count	Moderate local pain	++	[23, 25]
577‐nm PDL	disrupts sebaceous gland function; produces phototoxic effects to reduce *C. acnes* load	Significant (*p* < 0.05) reduction in inflammatory lesion count	Very mild local pain	++	[26]
Fractional					
1064‐nm Nd:YAG laser	Targets the vascular component of inflammatory acne, decreases the perifollicular stratum corneum and epithelium, and modulates cytokine release	Good reduction of inflammatory lesions and erythema	Localized discomfort; very few transient pigmentation changes	++	[18, 19, 21, 22, 24, 26]

*Note:* High quality (++++)— Further research is very unlikely to change confidence in the estimate of effect. Moderate quality (+++)— Further research is likely to have an important impact on confidence in the estimate of effect and may change the estimate. Low quality (++)— Further research is very likely to have an important impact on confidence in the estimate of effect and is likely to change the estimate. Very low quality (+)— Any estimate of effect is very uncertain.

### 3.1 Non‐Fractional Lasers

The 1450‐nm diode laser (DL), the 1540‐/1550‐nm Erbium: glass (Er:Glass) laser, and the 1726‐nm laser system have all been used to treat inflammatory acne lesions. They improve acne by reducing sebum production through photothermal mechanisms and by disrupting sebaceous gland function [29].

#### 3.1.1 1450‐nm Diode Lasers

A 1450‐nm diode laser is a long‐pulsed laser (LPL). LPL delivers selective photothermal energy to the sebaceous glands. The absorption of light by lipids generates localized heat, inducing coagulation and apoptosis of sebocytes, thereby reducing sebum production and preventing the formation of new acne lesions [30, 31].

Treatment with an infrared 1450‐nm diode laser with a dynamic cooling device has been demonstrated to safely and effectively reduce facial inflammatory acne lesions with fluences as high as 14 J/cm^2^ [32, 33].

#### 3.1.2 1540/1550‐nm Er:Glass Laser

1540‐ and 1550‐nm Er:Glass lasers are considered safe and effective options for treating both active acne lesions and acne scars [34]. 1540/1550‐nm Erbium:Glass laser therapy reduces facial skin oiliness accompanied by a significant decrease in sebaceous gland size, without any detectable morphological changes in the epidermis or dermis [13].

1540/1550‐nm Er:Glass laser has provided promising results in mild‐to‐moderate acne with laser settings including a fluence of 500–600 mJ/pulse, with three to four stacked pulses emitted at a rate of 2 Hz, and up to 2 passes per treatment session [14].

#### 3.1.3 1726‐nm Diode Lasers

The 1726‐nm diode laser is the most recently introduced non‐ablative laser and was approved by the FDA for the treatment of acne in 2022. It durably targets sebaceous gland activity through selective photothermolysis as this wavelength is only absorbed in sebum and preserves the dermis and epidermis structure [15, 16, 35, 36]. Because of the low absorption by skin pigmentation, 1726 nm lasers are considered effective and safe across all skin types [15, 35]. Diode lasers may be used in mild, moderate, and severe acne.

Recently, recommendations for the use of this type of non‐ablative LBT have been made available [37].

#### 3.1.4 Pulsed Dye Laser (PDL, 585–595 nm)

PDL alters sebaceous gland function through its photothermal effects on both the glands and the surrounding microvasculature [38, 39]. It targets hypervascularization associated with inflammation and promotes the expression of TGF‐β1, a key regulator involved in suppressing microcomedone formation and resolving inflammatory acne. As a result, PDL reduces the number of comedones and inflammatory lesions while it also improves acne‐related erythema [17, 39]. For PDL, typical protocols involve fluences of 7–10 J/cm^2^ with pulse durations of 0.45–1.5 ms, delivered in four to six sessions spaced two to 4 weeks apart [39].

Clinical investigations have demonstrated that PDL decreases inflammatory lesions, especially in patients with erythematous lesions [18, 19, 40]. However, the efficacy of PDL in the treatment of inflammatory acne remains to be confirmed through large, well‐designed clinical investigations [20, 39].

#### 3.1.5 Potassium Titanyl Phosphate 532‐Nm Laser

KTP laser is a photoangiolytic laser with a 532‐nm wavelength that is selectively absorbed by oxyhemoglobin [41]. In acne, KTP has anti‐inflammatory effects, reducing both erythema associated with inflammatory acne and sebum production [34, 42]. There is only limited evidence to support the use of KTP laser for active acne treatment and its effects on the microecosystem of sebaceous glands in acne lesions requiring further investigations [34].

### 3.2 Fractional Lasers

The Q‐switched Nd:YAG laser, with its long wavelength of 1064 nm, allows deep skin penetration with reduced absorption by epidermal melanin.

The 1064‐nm Nd:YAG laser reduces acne lesions by targeting the vascular component of inflammatory acne, decreasing the perifollicular stratum corneum and epithelium, and modulating cytokine release, specifically by increasing TGF‐β while lowering IL‐8 and TLR‐2 levels. Additionally, this laser system can significantly decrease sebum production, further contributing to its therapeutic effects in acne treatment [21, 34, 43].

The 1064 nm Nd:YAG laser is especially suited to patients with oily skin and moderate to severe acne resistant to topical therapy [18]. It is commonly applied with long‐pulsed settings of 40–50 J/cm^2^ and pulse durations of 15–30 ms for three to six sessions at two‐ to 4‐week intervals.

Several clinical studies have demonstrated that repeated sessions of the 1064‐nm Nd:YAG laser significantly decrease sebum excretion, while improving inflammatory and non‐inflammatory acne lesions with a lower risk of pigmentary changes in darker skin types [18, 22, 43, 44, 45, 46].

### 3.3 Combination Therapy

Acne therapy with topically applied gold microparticles followed by 1064‐nm Nd:YAG laser irradiation has been shown to be effective in moderate to moderately severe acne [47].

A second randomized, comparative study conducted in 13 subjects assessed the combined benefit of a 675‐nm laser and oral isotretinoin compared with the laser alone. Results showed an improved clinical outcome for the combination compared with the laser alone [48].

### 3.4 Current Evidence of Clinical Investigations

The authors analyzed 15 of the most pertinent clinical investigations using eight different fractional and non‐fractional non‐ablative LBT, which are the most frequently reported LBT used to treat active acne lesions [13, 14, 15, 16, 17, 18, 19, 20, 21, 22, 23, 24, 25, 26]. These lasers are listed in Table 1. However, the interpretation of these results is limited by small sample sizes, heterogeneous study designs, and variability in laser parameters.

Table 2 provides a condensed summary of the study results.

**TABLE 2:** Condensed summary table of characteristics and results of studies analyzed [13, 14, 15, 16, 17, 18, 19, 20, 21, 22, 23, 24, 25, 26].

Category	Details
Median age	Median: 21.5 years (range: 16–43)
Sex	50%–75% female
Skin phototype	11 studies: phototype II–IV 3 studies: phototype V 1 study: phototype VI
Affected area	14 studies: face 1 study: not specified
Quantification of area	Not specified
Acne severity (several grades possible)	9 studies: mild acne 12 studies: moderate acne; 5 studies: severe acne 3 studies: not quantified
Duration of acne	4 studies: 3–9 years 11 studies: not specified
Previous treatments (several treatments possible)	4 studies: topical treatments 10 studies: topical treatments 5 studies: not reported
Isotretinoin use	9 studies: yes 5 studies: no 1 study: not specified
Associated treatment with laser	1 study: systemic treatment; 2 studies: topical treatment (adapalene, tretinoin, or BPO) 6 studies: no associated treatment 6 studies: not specified
Study design	9 studies: split‐face randomized 6 studies: non‐comparative: 6 studies
LBT technology	Non‐fractional: 15 studies using 8 different LBT
Number of patients	Between 12 and 104
Number of centers	Single‐center studies: 14 (93%) Multicenter studies/y?: 1 (7%)
Non‐ablative LBT used (several LBT possible)	8 studies: PDL 6 studies: Nd:YAG 2 studies: 1726‐nm 2 studies: 1540‐nm
Number and frequency of sessions	3–4 sessions (range: 1–7 sessions)
Follow‐up duration	12 weeks
Primary outcome	13 studies: lesion count 1 study: acne severity score 1 study: combined outcome measures (lesions count and severity score)
Efficacy results (lesion count)	Reduction in lesions count Improvement versus baseline (no significant difference between groups)
Efficacy results (scales: GEA, IGA, etc.)	8 studies used validated scales; improvement vs. baseline (no difference between groups)
Side effects	Well tolerated: minimal adverse events with transient erythema Mild: erythema 100%, edema 29%, vesicles 29%, crusting 29%, hyperpigmentation 29%, transient flare‐up 18%, dryness 6%, paraesthesia 6% Mild to moderate pain; score 4.9/10 (without anesthesia) Superficial scars: 29%
Patient satisfaction	High satisfaction

Almost all studies were conducted at a single investigational site (14 single‐center studies and one multicenter study), nine used a split‐face design and six a non‐comparative design; ten evaluated the efficacy of non‐fractional lasers and five evaluated the efficacy a non‐fractional laser (1064 nm Nd:YAG laser). The studies included between 12 and 104 patients. PDL was the most frequently used LBT (eight studies), followed by Nd:YAG (6 studies) 1726 nm and 1540 nm (each two studies). Thirteen studies performed lesion counts, one used severity grading and another used both. The principal area treated was the face (14 studies), one study did not specify the target area. The average follow‐up was 12 weeks.

The median patient age was 21.5 years and 50%–75% of the patients were women. The main phototypes were II to IV in 11 studies; three reported phototype V, and only one study included, a few patients with phototype VI. Acne was mainly mild (nine subgroups) or moderate (12 subgroups); three subgroups included severe acne, and three studies did not report acne severity. Four studies reported a history of acne ranging from three to 9 years.

Ten studies reported previous topical treatments and five did not report any previous treatment. In nine studies, patients had previously received isotretinoin. Treatments associated with LBT included systemic treatment (one study), retinoids or benzoyl peroxide (two studies).

Globally, the studies reported a substantial reduction in lesion counts, mainly inflammatory lesions, ranging from a 46% reduction for a combination of PDL 589 nm/1319 nm and benzoyl peroxide after 12 weeks and four sessions every 2 weeks to a reduction of 92.9% for a combination of PDL (585 nm) and 1064 nm Nd:YAG after 12 weeks and four sessions every 2 weeks. A decrease of up to −70% in non‐inflammatory lesions after 12 weeks and four sessions 2 weeks apart was also observed. Pain was mainly mild to moderate, and patient satisfaction concerning the treatment outcome was high.

Based on these findings, the experts amended a previously proposed treatment algorithm (Figure 1) presented by a multidisciplinary working group on acne to incorporate non‐ablative LBT [49].

**FIGURE 1:**
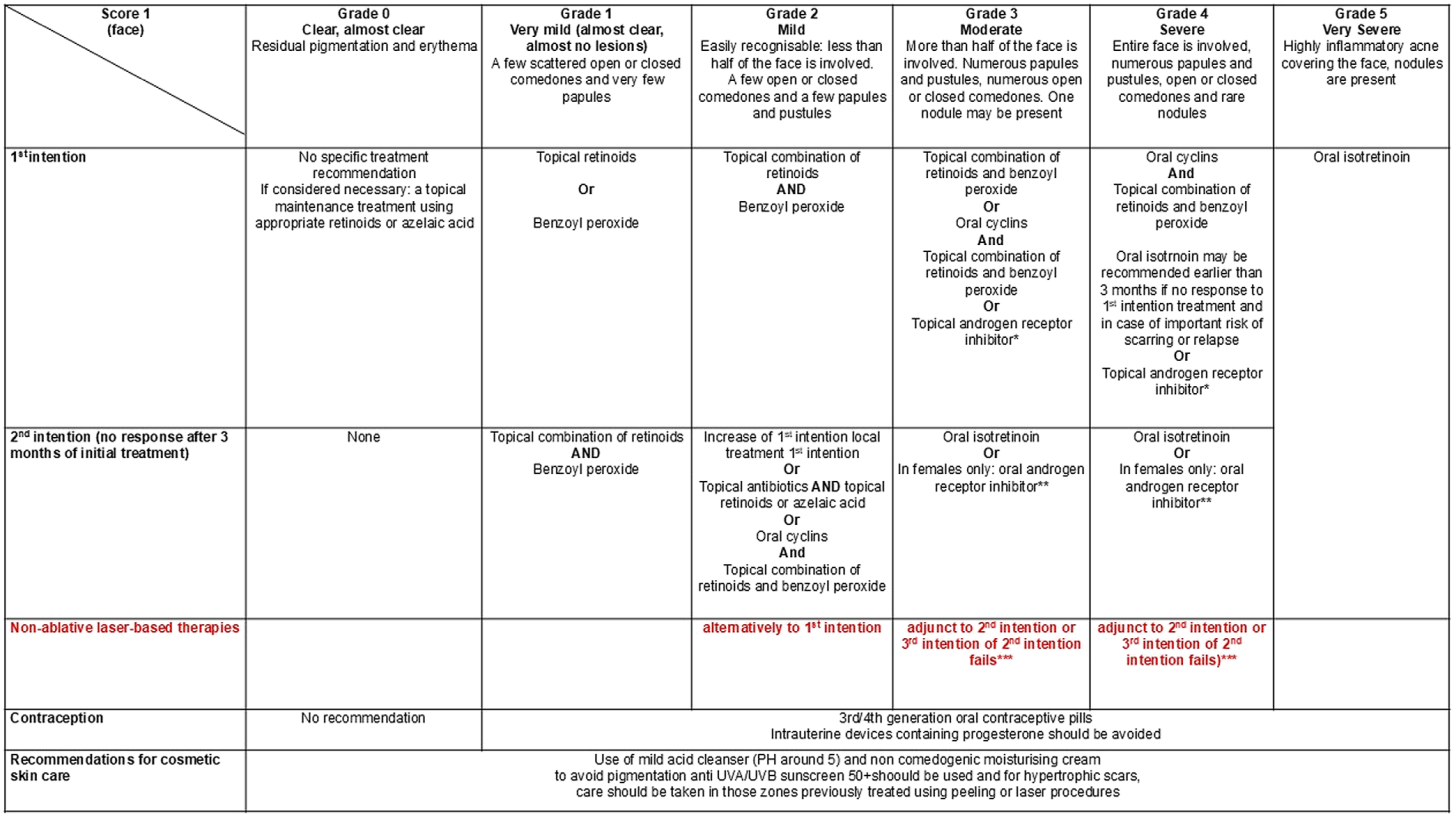
Amended active acne lesion treatment algorithm including non‐ablative LBT (based on Le Cleach et al. [49]). Topical retinoids: Adapalene, tazarotene, tretinoin, trifarorotene. Topical androgen receptor inhibitor: Clascosterone (Drugs Dermatol. 2024 Jan 1; 23 (1):1278–1283). Oral cyclins: Doxycyclin, tetracyclin. Oral androgen receptor inhibitor: Spironolactone (Acta Derm Venereol. 2024 Feb 21; 104: Adv26002. doi:10.2340/actadv.v104.26002), authorisation pending. *Where available.**Where authorized to treat active acne lesions. ***Not currently indicated in active acne lesions.

## 4 Discussion

Pharmacological treatments require long‐term use and high compliance to successfully treat acne. They may also cause local side effects such as erythema, skin dryness, burning, and pruritus, which are frequently the cause of poor treatment adherence [50]. Moreover, many of these acne treatments are not indicated in pregnant women. In this context, LBT may be considered a rapid, potentially beneficial and safe treatment alternative requiring no specific treatment adherence.

PDL directly targets *C. acnes* via the production of porphyrins. The efficacy of non‐ablative laser and light‐based therapies in acne is unlikely to be explained solely by their anti‐inflammatory properties. Available evidence suggests a dual mechanism involving both modulation of cutaneous inflammation and direct targeting of sebaceous glands. Histological studies have demonstrated structural alterations of sebaceous glands following treatment with the 1450‐nm diode laser, while selective photothermolysis of sebaceous glands has been even more clearly documented with the 1726‐nm laser [16, 29, 32, 51, 52]. Although less aggressive and not impacting the structure of the epidermis, non‐ablative LBT still may disturb the epidermal microbiome and the skin microbiome, especially when used repeatedly or at high intensities [53, 54, 55].

In the future, investigating the interactions between non‐ablative LBT and the epidermal and dermal skin microbiome, particularly its effects on two skin commensals *C. acnes* phylotype IA1 and *S. epidermidis* , will be important. Further research should also investigate how dermocosmetics may help maintain or prevent alterations of the skin barrier, lipid film, and key commensal bacteria following LBT. The analysis of the 15 clinical investigations showed that the efficacy and safety of non‐ablative LBT in the treatment of active acne lesions was globally good [13, 14, 15, 16, 17, 18, 19, 20, 21, 22, 23, 24, 25, 26]. Common side effects include transient erythema, edema, mild pain, and post‐treatment hyperpigmentation, especially in Fitzpatrick skin types IV–VI [56]. Pre‐ and post‐treatment care such as sun protection, cooling, and topical anti‐inflammatory agents have been suggested to minimize LBT‐related side effects.

However, currently published studies have limited methodological robustness, precluding evidence‐based recommendations. Direct comparisons with standard treatments such as isotretinoin or systemic antibiotics are lacking. Furthermore, the potential impact of laser‐based therapies on the skin microbiome and long‐term skin homeostasis remains insufficiently explored.

Finally, regardless of their potential clinical benefit, very little is known about the long‐term impact on the skin, its appendages, and microbiome requiring in‐depth research to confirm its inoffensiveness [57].

Standardization of laser protocols remains a major challenge. Variability in energy settings, wavelength selection, pulse duration, and session intervals across studies complicates comparisons and evidence synthesis. The analyzed studies were principally single‐center, split‐face or observational investigations, conducted in small patient populations and using different clinical parameters and scales to assess the efficacy of LBT. In the absence of standardized protocols and guidelines, personalized laser therapy integrating patient‐specific factors such as skin type, acne phenotype and severity, and genetic markers to optimize outcomes may help optimize treatment outcomes and limit laser‐associated risks and should therefore be further investigated. Furthermore, mechanistic studies investigating cellular and molecular responses to LBT may reveal novel targets to improve acne management. Finally, little is currently known about the long‐term impact of repeated laser sessions on the skin, its appendages and the skin microbiome.

Recently, the first recommendations for the use of 1726‐nm non‐ablative LBT in mild, moderate, and severe acne have been proposed [39]. However, these recommendations are limited to a one single type of laser and were financially supported by the manufacturer of a 1726‐nm non‐ablative LBT.

In 2026, the European Dermatology Forum released updated guidelines for the treatment of acne providing open recommendations (neither for nor against) for the use of laser in acne of any severity [7].

Despite the limited clinical data and the absence of independent recommendations on the use of non‐ablative LBT in the treatment of active acne lesions, the authors consider that non‐ablative LBT may be useful in mild acne and as an adjuvant second‐line treatment to conventional treatment or if conventional second‐line treatment fails and may also be useful in the treatment of acne scars and post‐inflammatory hyperpigmentation.

Thus, to provide dermatologists with minimum guidance on the use of non‐ablative LBT for treating patients with acne, the experts considered that incorporating non‐ablative LBT into a currently used treatment algorithm may help to better choose adapted treatments for each patient and to limit risks. To this end, the experts amended a currently used treatment algorithm (Figure 1) presented by a multidisciplinary working group on acne to incorporate non‐ablative LBT [49].

In conclusion, non‐ablative LBT may be a useful and effective option for acne management, offering both lesion clearance and cosmetic improvement. However, proper protocol selection and validated clinical investigations in large cohorts that allow for a critical assessment of the different available laser‐based therapies are required to objectively confirm its clinical benefit in the treatment of active acne lesions.

## Author Contributions

Conceptualization: Fabienne Ballanger, Marie‐Thérèse Leccia, Nicole Auffret, Jean‐Paul Claudel, Brigitte Dréno; Data curation: Not applicable; Formal analysis: Not applicable; Funding acquisition: Fabienne Ballanger, Marie‐Thérèse Leccia, Nicole Auffret, Jean‐Paul Claudel, Brigitte Dréno; Investigation: Not applicable; Methodology: Fabienne Ballanger, Marie‐Thérèse Leccia, Nicole Auffret, Jean‐Paul Claudel, Brigitte Dréno; Project administration: Brigitte Dréno; Resources: Fabienne Ballanger, Marie‐Thérèse Leccia, Nicole Auffret, Jean‐Paul Claudel, Brigitte Dréno; Software: Not applicable; Supervision: Brigitte Dréno; Validation: Fabienne Ballanger, Marie‐Thérèse Leccia, Nicole Auffret, Jean‐Paul Claudel, Brigitte Dréno; Visualization: Fabienne Ballanger, Marie‐Thérèse Leccia, Nicole Auffret, Jean‐Paul Claudel, Brigitte Dréno; Writing – original draft: Fabienne Ballanger, Marie‐Thérèse Leccia, Nicole Auffret, Jean‐Paul Claudel, Brigitte Dréno; Writing – review and editing: Fabienne Ballanger, Marie‐Thérèse Leccia, Nicole Auffret, Jean‐Paul Claudel, Brigitte Dréno.

## Funding

The work of this expert group is supported by Galderma International, France.

## Conflicts of Interest

The expert board is supported by Galderma International, France.

## Data Availability

The data that support the findings of this study are available from the corresponding author upon reasonable request.

## References

[1] Y. Deng , F. Wang , and L. He , “Skin Barrier Dysfunction in Acne Vulgaris: Pathogenesis and Therapeutic Approaches,” Medical Science Monitor 30 (2024): e945336. 10.12659/MSM.945336 PMC11650898 39668545

[2] B. Dréno , M. A. Dagnelie , A. Khammari , and S. Corvec , “The Skin Microbiome: A New Actor in Inflammatory Acne,” American Journal of Clinical Dermatology 21, no. Suppl 1 (2020): 18–24. 10.1007/s40257-020-00531-1 PMC7584556 32910436

[3] C. C. Zouboulis , T. Coenye , L. He , et al., “Sebaceous Immunobiology ‐ Skin Homeostasis, Pathophysiology, Coordination of Innate Immunity and Inflammatory Response and Disease Associations,” Frontiers in Immunology 13 (2022): 1029818. 10.3389/fimmu.2022.1029818 PMC9686445 36439142

[4] M. A. Dagnelie , E. Montassier , A. Khammari , C. Mounier , S. Corvec , and B. Dréno , “Inflammatory Skin Is Associated With Changes in the Skin Microbiota Composition on the Back of Severe Acne Patients,” Experimental Dermatology 28, no. 8 (2019): 961–967. 10.1111/exd.13988 31173650

[5] A. L. Zaenglein , A. L. Pathy , B. J. Schlosser , et al., “Guidelines of Care for the Management of Acne Vulgaris,” Journal of the American Academy of Dermatology 74, no. 5 (2016): 945–973.e33. 10.1016/j.jaad.2015.12.037 26897386

[6] A. Nast , B. Dreno , V. Bettoli , et al., “European Evidence‐Based (S3) Guideline for the Treatment of Acne ‐ Update 2016 ‐ Short Version,” Journal of the European Academy of Dermatology and Venereology 30, no. 8 (2016): 1261–1268. 10.1111/jdv.13776 27514932

[7] A. Nast , B. H. Al Wattar , M. Beylot Barry , et al., “Update of the EuroGuiDerm Evidence‐Based Guideline for the Treatment of Acne‐Short Version,” Journal of the European Academy of Dermatology and Venereology 40 (2026): 1162–1172. 10.1111/jdv.70331 PMC13308670 41847993

[8] K. A. Habeshian and B. A. Cohen , “Current Issues in the Treatment of Acne Vulgaris,” Pediatrics 145, no. Suppl 2 (2020): S225–S230. 10.1542/peds.2019-2056L 32358215

[9] H. Xu and H. Li , “Acne, the Skin Microbiome, and Antibiotic Treatment,” American Journal of Clinical Dermatology 20, no. 3 (2019): 335–344. 10.1007/s40257-018-00417-3 PMC6534434 30632097

[10] L. E. Wiznia , M. L. Stevenson , and A. R. Nagler , “Laser Treatments of Active Acne,” Lasers in Medical Science 32, no. 7 (2017): 1647–1658. 10.1007/s10103-017-2294-7 28776112

[11] L. Ishii , S. Deoghare , and M. Boen , “Light and Laser‐Based Therapy in Treatment of Acne Vulgaris: A Clinical Review,” Journal of the American Academy of Dermatology 93, no. 4 (2025): 1058–1064. 10.1016/j.jaad.2025.06.046 40550334

[12] J. Howick , I. Chalmers , P. Glasziou , et al., “Oxford Centre for Evidence‐Based Medicine 2011 Levels of Evidence,” (2011), https://www.cebm.net/wp‐content/uploads/2014/06/CEBM‐Levels‐of‐Evidence‐21%20pdf.

[13] Y. Politi , A. Levi , I. Snast , E. D. Ad , and M. Lapidoth , “Integrated Cooling‐Vacuum‐Assisted Non‐Fractional 1540‐Nm Erbium:Glass Laser: A New Modality for the Simultaneous Effective Treatment of Acne Lesions and Scars,” Journal of Drugs in Dermatology 17, no. 11 (2018): 1173–1176. 30500135

[14] Y. Politi , A. Levi , C. D. Enk , and M. Lapidoth , “Integrated Cooling‐Vacuum‐Assisted 1540‐Nm Erbium:Glass Laser Is Effective in Treating Mild‐To‐Moderate Acne Vulgaris,” Lasers in Medical Science 30, no. 9 (2015): 2389–2393. 10.1007/s10103-015-1816-4 26428932

[15] M. Alexiades , A. Kothare , D. Goldberg , and J. S. Dover , “Novel 1726 Nm Laser Demonstrates Durable Therapeutic Outcomes and Tolerability for Moderate‐To‐Severe Acne Across Skin Types,” Journal of the American Academy of Dermatology 89, no. 4 (2023): 703–710. 10.1016/j.jaad.2023.05.085 37328000

[16] D. Goldberg , A. Kothare , M. Doucette , et al., “Selective Photothermolysis With a Novel 1726 Nm Laser Beam: A Safe and Effective Solution for Acne Vulgaris,” Journal of Cosmetic Dermatology 22, no. 2 (2023): 486–496. 10.1111/jocd.15602 36576854

[17] R. Bencharattanaphakhi , S. Wananukul , T. Tempark , and S. Chatproedprai , “A 595 Nm Pulsed Dye Laser as an Adjuvant Intervention for Post‐Comedone Extraction Erythema and Comedone Reduction: A Randomized, Split‐Face Controlled Trial,” Journal of Cosmetic Dermatology 23, no. 5 (2024): 1645–1653. 10.1111/jocd.16178 38192163

[18] N. Chalermsuwiwattanakan , S. Rojhirunsakool , N. Kamanamool , S. Kanokrungsee , and M. Udompataikul , “The Comparative Study of Efficacy Between 1064‐Nm Long‐Pulsed Nd:YAG Laser and 595‐Nm Pulsed Dye Laser for the Treatment of Acne Vulgaris,” Journal of Cosmetic Dermatology 20, no. 7 (2021): 2108–2115. 10.1111/jocd.13832 33226176

[19] M. M. Salah El Din , N. A. Samy , and A. E. Salem , “Comparison of Pulsed Dye Laser Versus Combined Pulsed Dye Laser and Nd:YAG Laser in the Treatment of Inflammatory Acne Vulgaris,” Journal of Cosmetic and Laser Therapy 19, no. 3 (2017): 149–159. 10.1080/14764172.2016.1262962 27911144

[20] R. Lekwuttikarn , T. Tempark , S. Chatproedprai , and S. Wananukul , “Randomized, Controlled Trial Split‐Faced Study of 595‐Nm Pulsed Dye Laser in the Treatment of Acne Vulgaris and Acne Erythema in Adolescents and Early Adulthood,” International Journal of Dermatology 56, no. 8 (2017): 884–888. 10.1111/ijd.13631 28447362

[21] T. M. Hammoda , N. A. Ahmed , and M. Hamdino , “Fractional CO(2) Laser Versus 1064‐Nm Long‐Pulsed Nd:YAG Laser for Inflammatory Acne Vulgaris Treatment: A Randomized Clinical Trial,” Lasers in Medical Science 38, no. 1 (2023): 187. 10.1007/s10103-023-03855-6 PMC10435603 37592125

[22] A. M. Ibrahim , G. A. B. Omar , and M. Hamdino , “Long‐Pulsed Nd: YAG Laser (1064 Nm) Versus Intralesional Botulinum Toxin Type (A) in Acne Vulgaris Therapy: A Split Face Study,” International Journal of Dermatology 62, no. 6 (2023): 822–830. 10.1111/ijd.16519 36468835

[23] S. Boonpethkaew , Y. Ratanapokasatit , S. Chirasuthat , and P. Wattanakrai , “Efficacy and Safety of the 589/1319 Nm Solid‐State Dual‐Wavelength Laser Combined With Topical Benzoyl Peroxide for Inflammatory Acne Vulgaris: A Split‐Face Randomized Controlled Trial,” Archives of Dermatological Research 317, no. 1 (2025): 635. 10.1007/s00403-025-04146-6 PMC11947063 40140055

[24] D. Maghsoodloo , A. Sadeghzadeh‐Bazargan , M. Ghassemi , and A. Goodarzi , “Comparison of the Efficacy of Q‐Switched Versus Long‐Pulsed Fractional 1064 Nm Nd: YAG Laser in the Treatment of Active Facial Acne: A Double‐Blind, Randomized, Controlled, Split‐Face Clinical Trial,” Health Science Reports 9, no. 4 (2026): e72354. 10.1002/hsr2.72354 PMC13077659 41988396

[25] J. Lim , “Efficacy and Safety of Solid‐State Dual‐Wavelength Lasers for the Treatment of Moderate‐To‐Severe Inflammatory Acne in Asian Populations,” Plastic and Reconstructive Surgery. Global Open 12 (2024): e5550. 10.1097/GOX.0000000000005550 PMC10817014 38288419

[26] E. M. Mohamed , H. L. Abdel‐Aleem , G. M. H. Elazab , and M. A. Rageh , “Comparative Efficacy and Safety of 577‐Nm Diode Laser Versus 1064‐Nm Nd: YAG Laser for Inflammatory Acne Vulgaris: A Split‐Face Randomized Study,” Lasers in Medical Science 40, no. 1 (2025): 422. 10.1007/s10103-025-04654-x PMC12513887 41071332

[27] M. R. Alexiades‐Armenakas , J. S. Dover , and K. A. Arndt , “The Spectrum of Laser Skin Resurfacing: Nonablative, Fractional, and Ablative Laser Resurfacing,” Journal of the American Academy of Dermatology 58, no. 5 (2008): 719–737. 10.1016/j.jaad.2008.01.003 18423256

[28] Y. Wang , S. Kuo , M. Shu , et al., “ *Staphylococcus epidermidis* In the Human Skin Microbiome Mediates Fermentation to Inhibit the Growth of *Propionibacterium acnes* : Implications of Probiotics in Acne Vulgaris,” Applied Microbiology and Biotechnology 98, no. 1 (2014): 411–424. 10.1007/s00253-013-5394-8 PMC3888247 24265031

[29] J. Xu , S. Huang , Z. Fu , et al., “Effects of Light and Laser Therapies on the Microecosystem of Sebaceous Glands in Acne Treatment,” Photodermatology, Photoimmunology & Photomedicine 41, no. 1 (2025): e70005. 10.1111/phpp.70005 39754335

[30] H. H. Kwon , S. C. Choi , J. Y. Jung , Y. I. Bae , and G. H. Park , “Comparison of Novel Dual Mode vs Conventional Single Pass of a 1450‐Nm Diode Laser in the Treatment of Acne Vulgaris for Korean Patients: A 20‐Week Prospective, Randomized, Split‐Face Study,” Journal of Cosmetic Dermatology 17, no. 6 (2018): 1063–1068. 10.1111/jocd.12788 30225903

[31] Y. H. Cho , S. M. Oh , J. Y. Choi , and K. H. Jeong , “Acne Treatment Based on Selective Photothermolysis With Topically Delivered Light‐Absorbing Platinum Nanoparticles,” Lasers in Medical Science 38, no. 1 (2023): 125. 10.1007/s10103-023-03787-1 37209256

[32] P. M. Friedman , M. H. Jih , A. Kimyai‐Asadi , and L. H. Goldberg , “Treatment of Inflammatory Facial Acne Vulgaris With the 1450‐Nm Diode Laser: A Pilot Study,” Dermatologic Surgery 30, no. 2 Pt 1 (2004): 147–151. 10.1111/j.1524-4725.2004.30062.x 14756641

[33] S. Q. Wang , J. T. Counters , M. E. Flor , and B. D. Zelickson , “Treatment of Inflammatory Facial Acne With the 1,450 Nm Diode Laser Alone Versus Microdermabrasion Plus the 1,450 Nm Laser: A Randomized, Split‐Face Trial,” Dermatologic Surgery 32, no. 2 (2006): 249–255. 16442046

[34] M. K. Li , C. Liu , and J. T. S. Hsu , “The Use of Lasers and Light Devices in Acne Management: An Update,” American Journal of Clinical Dermatology 22, no. 6 (2021): 785–800. 10.1007/s40257-021-00624-5 34287769

[35] J. Bittar , P. Hooper , and J. S. Dover , “1726 Nm Lasers for the Treatment of Acne Vulgaris,” Skin Therapy Letter 29, no. 1 (2024): 5–7. 38271552

[36] M. G. Scopelliti , A. Kothare , and M. Karavitis , “A Novel 1726‐Nm Laser System for Safe and Effective Treatment of Acne Vulgaris,” Lasers in Medical Science 37, no. 9 (2022): 3639–3647. 10.1007/s10103-022-03645-6 36197599

[37] A. Metelitsa , R. S. Batra , A. Chapas , J. Fromowitz , and E. Graber , “Consensus Panel Recommendations for Optimizing Use of a Contact‐Cooled 1726 Nm Laser for the Treatment of Acne,” Journal of Cosmetic Dermatology 25, no. 2 (2026): e70699. 10.1111/jocd.70699 PMC12856072 41612575

[38] E. Forbat and F. Al‐Niaimi , “Nonvascular Uses of Pulsed Dye Laser in Clinical Dermatology,” Journal of Cosmetic Dermatology 18, no. 5 (2019): 1186–1201. 10.1111/jocd.12924 31002479

[39] T. H. Yang , C. N. Li , and Y. C. Huang , “The Efficacy of Pulsed Dye Laser Treatment for Acne Vulgaris: A Systemic Review and Meta‐Analysis,” Dermatologic Surgery 48, no. 2 (2022): 209–213. 10.1097/DSS.0000000000003345 34923532

[40] N. Voravutinon , J. Rojanamatin , D. Sadhwani , S. Iyengar , and M. Alam , “A Comparative Split‐Face Study Using Different Mild Purpuric and Subpurpuric Fluence Level of 595‐Nm Pulsed‐Dye Laser for Treatment of Moderate to Severe Acne Vulgaris,” Dermatologic Surgery 42, no. 3 (2016): 403–409. 10.1097/DSS.0000000000000658 26890802

[41] J. Ma , R. Fang , R. Zhen , et al., “A 532‐Nm KTP Laser for Vocal Fold Polyps: Efficacy and Relative Factors,” Ear, Nose, & Throat Journal 100, no. 1_suppl (2021): 87s–93s. 10.1177/0145561320946153 32776834

[42] N. S. Sadick and A. Cardona , “Laser Treatment for Facial Acne Scars: A Review,” Journal of Cosmetic and Laser Therapy 20, no. 7–8 (2018): 424–435. 10.1080/14764172.2018.1461230 30395754

[43] K. Kesty and D. J. Goldberg , “650 Usec 1064nm Nd:YAG Laser Treatment of Acne: A Double‐Blind Randomized Control Study,” Journal of Cosmetic Dermatology 19, no. 9 (2020): 2295–2300. 10.1111/jocd.13480 32447830

[44] H. H. M. Eltanany , F. Qawy , M. Hamdino , and M. S. Ali , “A Split Face Study Comparing Intralesional Tranexamic Acid Versus 1064 Nm Long Pulsed Nd: YAG Laser for Acne Vulgaris Treatment,” Archives of Dermatological Research 317, no. 1 (2025): 709. 10.1007/s00403-025-04164-4 40221536

[45] O. Artzi , A. Koren , W. Shehadeh , and O. Friedman , “Quasi Long‐Pulsed 1064nm Nd:YAG (Micro Pulsed) Technology for the Treatment of Active Acne: A Case Series,” Journal of Cosmetic Dermatology 20, no. 7 (2021): 2102–2107. 10.1111/jocd.14128 33834583

[46] N. H. Moftah , A. M. Mansour , and S. M. A. Ibrahim , “Clinical Evaluation of Efficacy of Intralesional Platelet‐Rich Plasma Injection Versus 1064 Nm Long‐Pulsed Neodymium:YAG Laser in the Treatment of Inflammatory Acne Vulgaris in Adolescent and Post‐Adolescent Patients: A Prospective Randomized Split‐Face Comparative Study,” Lasers in Medical Science 37, no. 5 (2022): 2471–2478. 10.1007/s10103-022-03510-6 PMC9232433 35084634

[47] J. Waibel , E. Graber , T. Lain , A. Gewirtzman , C. Feser , and W. Werschler , “A Prospective, Multicenter, Study of Laser‐Activated Gold Microparticles for Treating Patients Using Concomitant Topical Retinoids for Mild‐To‐Moderate Inflammatory Acne,” Journal of Drugs in Dermatology 22, no. 11 (2023): 1088–1094. 10.36849/JDD.1088 37943275

[48] A. Haus , A. Clementi , G. Cannarozzo , et al., “Combining 675 Nm Laser With Isotretinoin for Enhanced Acne Vulgaris Treatment Outcomes,” Healthcare (Basel) 13, no. 23 (2025): 3068. 10.3390/healthcare13233068 PMC12692384 41373285

[49] L. Le Cleach , B. Lebrun‐Vignes , A. Bachelot , et al., “Guidelines for the Management of Acne: Recommendations From a French Multidisciplinary Group,” British Journal of Dermatology 177, no. 4 (2017): 908–913. 10.1111/bjd.15843 29052890

[50] B. Dréno , D. Thiboutot , H. Gollnick , et al., “Large‐Scale Worldwide Observational Study of Adherence With Acne Therapy,” International Journal of Dermatology 49, no. 4 (2010): 448–456. 10.1111/j.1365-4632.2010.04416.x 20465705

[51] M. H. Jih , P. M. Friedman , L. H. Goldberg , M. Robles , A. S. Glaich , and A. Kimyai‐Asadi , “The 1450‐Nm Diode Laser for Facial Inflammatory Acne Vulgaris: Dose‐Response and 12‐Month Follow‐Up Study,” Journal of the American Academy of Dermatology 55, no. 1 (2006): 80–87. 10.1016/j.jaad.2006.02.018 16781297

[52] D. Goldberg , S. Ronan , A. Bhatia , et al., “Safe and Effective Acne Treatment Across Skin Types With a 1726 Nm Sebum‐Selective Laser: One Year Data From a Prospective Multicenter Study,” Journal of the American Academy of Dermatology 94, no. 2 (2026): 517–524. 10.1016/j.jaad.2025.09.077 41052654

[53] J. R. Ellis , E. J. Powell , L. M. Tomasovic , et al., “Changes in the Skin Microbiome Following Dermatological Procedures: A Scoping Review,” Applied Microbiology 4, no. 2 (2024): 972–985. 10.3390/applmicrobiol4020066 PMC12994279 41853703

[54] R. C. de Sica , C. J. Rodrigues , D. A. Maria , and L. C. Cuce , “Study of 1550‐Nm Erbium Glass Laser Fractional Non‐Ablative Treatment of Photoaging: Comparative Clinical Effects, Histopathology, Electron Microscopy, and Immunohistochemistry,” Journal of Cosmetic and Laser Therapy 18, no. 4 (2016): 193–203. 10.3109/14764172.2015.1114645 26734913

[55] D. Haykal and M. Rocha , “Navigating the Intersection of Lasers and the Skin Microbiome: A New Frontier in Esthetic Dermatology,” Journal of Cosmetic Dermatology 24, no. 4 (2025): e70150. 10.1111/jocd.70150 PMC11959326 40165601

[56] K. Huerth , C. Ohanenye , A. Quartey , B. Jackson , and V. Callender , “Established and Emerging Laser Treatments for Acne Vulgaris in Diverse Skin Types,” Journal of Clinical and Aesthetic Dermatology 18, no. 6 (2025): 41–48. PMC12189219 40575606

[57] S. Angel , D. Boineau , S. Dahan , and S. Mordon , “Treatment of Active Acne With an er:Glass (1.54 μm) Laser: A 2‐Year Follow‐Up Study,” Journal of Cosmetic and Laser Therapy 8, no. 4 (2006): 171–176. 10.1080/14764170600915985 19839169

